# Abstract of the Proceedings of the Buffalo Medical Association

**Published:** 1857-08

**Authors:** Sanford B. Hunt


### ART. III.—*Abstract of the Proceedings of the Buffalo Medical Association.*

TUESDAY EVENING, July 7, 1857.

The association met.

Present—The President, Dr. Flint, in the chair. Drs. Wilcox, Miner, Hamilton, Rogers, Dayton, Strong, Newman, Hunt, Hutchins, Lemon, Gould, and Wyckoff.

The reading of the minutes was deferred.

Drs. Austin Flint, Jr., Sylvester Rankin, and —— Cole, were admitted to membership in this association.

*Prof. Flint*, from the committee on epidemic pharyngitis, reported that they had nothing new to bring forward.

*Prof. Hamilton* presented the following specimen:

A specimen of a "colloid cancer," removed from the upper jaw of a woman 25 years old, June 30th, 1857.

The disease had been progressing several years, and at the time of the removal it occupied the right antrum completely, and having involved all of the lower and lateral walls, it protruded far into the mouth, and outward upon the face. The patient had a pale and unhealthy complexion.

An incision having been made so as to lay bare the whole wall of the tumor, the mass was removed entire by the bone-forceps and a strong pair of scissors. The hæmorrhage was profuse, but only the palatine artery required a ligature.

The interior presents the characteristic appearance. The bone composing the upper jaw has been not simply infiltrated, but absorbed, and superseded by the new material. In consistence it resembles jelly, and is of a light straw color, with a tinge of green. Pressing upon it, an abundant serous fluid escapes. Fibrous septa are perceptible even to the naked eye; but submitted to the microscope large bundles of fibres are seen, resembling the white inelastic tissue, with an occasional yellow, elastic fibre, which latter Lebert remarks are sometimes found in these growths. Dr. Dalton, with Dr. Austin Flint, Jr., to whom I submitted the specimen for examination, found also a number of nucleated cancer cells.

I have three times before met with this form of cancer. In Feb., 1851, I removed a tumor of this character from the vulva of a lady, then about 25 years old. It had been growing several years, and latterly the growth had been very rapid. The tumor was of the size of a large cocoa nut, and hung from the right labium, attached only by a narrow pedicle, and weighing nearly two pounds. It was not tender or painful. I removed it completely with the knife.

On examination of its internal structure, I found it composed of a fibrous structure, with here and there portions converted into a gelatiniform mass, and of a pale yellow, green and pink color.

Six months from this date she thought the tumor had again commenced growing. It was now occasionally sore and a little painful. I have never heard from her since.

Jan. 29, 1851, assisted by Dr. Mixer, I removed a colloid cancer from J. K., æt. 47. The tumor had formed either in one of the glands or in the areolar tissue, of the right groin. At first it had grown slowly, but rapidly during the last year. It was smooth, irregularly round, and elastic, and measured ten inches in diameter at the base. His health was considerably impaired.

On examination after removal, I found almost the whole of this mass gelatiniform, and iridescent, the color changing from yellow to green, according as the light was reflected from it. The green, however, predominated. It weighed three pounds and a quarter.

The wound healed rapidly, but, about two years after, it again began to return, and in a few months was as large as before, and, having ulcerated, it threw out a prolific fungus and bled often.

Since then I have lost sight of him.

June 28, 1851, assisted by Dr. Boardman, I removed a uteaine polypus from a lady, æt. 44. The tumor was at that time projecting eight inches beyond the vulva, and as large as a child's head at nine months. It was smooth and elastic, being covered with a firm fibrous investment. We applied a strong ligature to its pedicle just within the os tincæ, and left it to slough off. The separation was soon accomplished, and on examination of the tumor I found it to be composed of a fibrous stroma, with large masses of colloid structure interposed. The color of these masses was a light straw. Its weight was one pound and a-half.

The tumor soon returned, and after a second removal she rapidly sank and died: having survived the first operation only about one month.

Such have been the results thus far in my experience of this class of growths, and whether they are entitled to rank as cancers or not, they seem to possess a degree of malignancy scarcely inferior to anv other forms of tumor.*

*Prof. Hamilton* then exhibited a specimen of fracture of the cricoid and thyroid cartilages, a description of which was published in the last number of the Buffalo Medical Journal.

He next produced a specimen of fracture of the neck of the femur within the capsule, for the privilege of presenting which he was indebted to the kindness of Prof. Crosby, of Dartmouth College. The patient, a lady, after meeting with the accident, recovered so as to be able to walk very well; but her case was one of very difficult diagnosis. She was seen by many surgeons, some of whom considered it a dislocation, others a fracture of the neck, and others still a fracture of the rim of the acetabulum. A post-mortem examination revealed the condition here presented. The neck was almost entirely absorbed, the opposing surfaces of the fracture manifesting no attempt at union, and the surfaces laminated and eburnated.

Prof. Hamilton commented at some length on the probabilities of a union within the capsule. He said that there were eleven specimens, claimed to be unions within the joint, now in the United States. Of these he had seen about half, and he would say that none of them were indubitable. Some of them might be, but none of them could be conclusively proved to be unions within the capsule.

Prof. Hamilton also presented a specimen of a foreign body removed from the knee-joint by Dr. Crosby. It was the segment of a sphere of bone evidently a portion of the external condyle of the femur somewhat eburnated by long friction. A young man suffered a severe wrench of the joint, and subsequently suffered repeated attacks of pain. Prof. Crosby removed the foreign body exhibited, by operation. The dimensions of the fragment were as follows: vertical diameter, one inch and five-eighths; left border half an inch in thickness, decreasing toward the right border. The case is remarkable as showing that a portion of the condyle of the femur can be fractured into the knee-joint, removed by the knife, and the motion of the joint preserved.

*Dr. Strong* gave a history of a case in which he diagnosticated Bright's Disease in a woman about to be confined. She had extensive œdema of

---

* Note. The three last cases have been previously reported in the tables furnished by Dr. Hamilton to Dr. Gross' paper, on the "Results of Surgical Operations in Malignant Diseases," and published in the Transactions of the Amer. Med. Association for 1853.

the limbs and puffiness of the face; the urine was albuminous. This lady has since been confined without untoward symptoms. The probability was that she would have had puerperal convulsions. That she did not, Dr. Strong was inclined to attribute to the fact that chloroform was administered during the labor, not to entire anæsthesia, but to a sufficient extent to modify the pains.

*Dr. Hutchins* inqnired if Dr. Strong considered albuminuria, during pregnancy, as an iufallible premonitory sign of puerperal convulsions?

*Dr. Strong* replied that he did not, but when the symptoms were as prominent as in this case, and as strongly indicative of Bright's disease, he should feel anxious about his patient. The œdema was extensive in the limbs and in the face. She had also considerable impairment of vision.

*Prof. Hamilton* inquired if the President had met with Dr. Isaacs' recent paper, in which he states that a healthy human kidney was rarely met with?

*The President* had not, and could not himself answer as to the justice of the statement.

*Dr. Miner* asked if, supposing that the case was admitted to be Bright's disease, with considerable anasarca, would there be from that fact alone any sufficient reason for apprehending puerperal convulsions during confinement?

*Dr. Strong* thought there would. In reply to further questions he stated that he did not express any positive opinion as to the effect of the chloroform.

*Dr. Newman* remarked that since Dr. Simpson had called attention to these cases, it had become evident that albuminuria was much more frequent in pregnant women than it had heretofore been supposed to be, though usually it resulted from congestion and not from fatty degeneration. Authors nearly all now took the view that convulsions were likely to occur in such cases from the poison in the blood, and patients should be carefully watched even for a week or ten days after the event of labor.

*Dr. Hutchins* had seen, within the last month, six cases of an eruptive disease, four of them in the same family. One of them was unquestionably measles, the others had a vesicular eruption, with pustules containing fluid. All of these cases had some bronchial affection and sore throat. He was undecided as to the character of these cases.

He had also seen four cases of what the patients had supposed to be some disease of the kidneys, manifested by considerable pain. No albumen was present. On applying nitric acid the urine became very red, much lime was present under the microscope. There was also a slight cheesy discharge from the urethra. There was no reason to suppose any venereal cause in these cases. They all recovered within three or four weeks.

*Dr. Newman* proposed Drs. C. B. Fanner and Henry Nichell for membership in this association.

*Dr. Wilcox* moved that a committee of three be appointed to report on the subject of albuminuria in pregnancy.

Drs. Newman, Strong and Hutchins, were appointed such committee.

*Prof. Hamilton* moved that Dr. Miner be instructed to report on the subject of dissection wounds, at the next meeting.

*Dr. Rogers* reported the case of Dr. Ebenezer B. Pulling, who assisted him in the examination of a woman who died of hæmorrhage. In the course of a few hours Dr. Pulling complained of pain in a finger which he had wounded. From a point in the finger the swelling spread to midway the arm above the elbow, and was there arrested. Soon after, however, the swelling commenced on the same finger of the other hand, and spread thence almost over the whole body. It proved fatal, and this case was the beginning of a fatal epidemic of erysipelas, the first case of which was a clergyman who had acted as a nurse to Dr. Pulling.

*Prof. Flint* presented a practical point. Dr. Ware, of Boston, had recently inquired if the profession here used whisky in tuberculosis. He said that the profession in Boston preferred whisky to other stimulants, and the coarser kinds of whisky in preference to better varieties. This fact is attributed to the greater quantity of fusel oil in that liquor. Dr. Flint had used other forms of stimulus, but mentioned cases in which whisky had seemed to be very beneficial.

*Prof. Hamilton* thought that whisky was the probable cause of sore legs and ulcers among the Irish.

Section 5, Chapter II, of the By-laws, was amended by striking out the words "two dollars on" from the last sentence of the by-laws.

And the association then adjourned.

SANFORD B. HUNT, Secretary.

